# An Atypical Aquaporin 1 Derived From *Paramecium multimicronucleatum* Functions as a Multifunctional Channel That Permeates Water, Glycerol, and Urea

**DOI:** 10.1111/jeu.70108

**Published:** 2026-07-03

**Authors:** Masaki Ishida, Shuichi Ueno, Takashi Tominaga, Kurumi Tanaka, Akito Horikawa, Manabu Hori

**Affiliations:** ^1^ School of Science Education Nara University of Education Nara Japan; ^2^ Graduate School of Sciences and Technology for Innovation Yamaguchi University Yamaguchi Japan; ^3^ Institute of Neuroscience, Tokushima Bunri University Kagawa Japan

**Keywords:** a*moeba*, aquaglyceroporin, *Dictyostelium*, mEGFP, NPA‐NPG motif, parasitic protist, super‐aquaporin, water and solute permeability

## Abstract

Although it had been reported that the translation product of the functional aquaporin gene, *aqp1,* is localized to the contractile vacuole complex of *Paramecium multimicronucleatum*, the molecules that pass through this channel protein had not been identified. In the present study, we introduced and expressed this protein in *Xenopus* oocytes and identified the molecules that pass through this channel protein using a swelling assay. The *aqp1* mRNA injected into *Xenopus* oocytes was found to be sufficiently expressed and functional within the oocytes. As a result, *P. multimicronucleatu*m AQP1 (PmAQP1) allowed water to pass through at a relatively slow rate of approximately 50 × 10^−4^cm/s, a rate comparable to that of aquaporins present in the contractile vacuoles of other protists. This water permeability was almost completely inhibited by tetraethylammonium, an orthodox aquaporin inhibitor, and phloretin, an aquaglyceroporin inhibitor. PmAQP1 also had the ability to pass glycerol or urea through, but the rates of these processes could not be accurately measured in this study. These findings demonstrate that PmAQP1 is an atypical multifunctional channel, suggesting that the contractile vacuole of free‐living protists plays a crucial role not only in osmoregulation but also in the clearance of metabolic wastes.

Aquaporins (AQPs) are a family of water channels widely distributed throughout living organisms and are broadly classified into three groups. The first group consists of “orthodox aquaporins” (AQP0, AQP1, AQP2, AQP4, AQP5, AQP6, and AQP8), which exhibit specific permeability to water molecules. The second group consists of “aquaglyceroporins” (AQP3, AQP7, AQP9, and AQP10), which, in addition to water, allow the passage of uncharged small‐molecule solutes such as glycerol (Fu et al. 2000), urea (Echevarria et al. 1994), ammonia (Holm et al. 2005), and even gases (Cooper and Boron 1998). The third type is called “super‐aquaporins” or “unorthodox aquaporins” (AQP11, AQP12). They are localized in intracellular compartments such as the endoplasmic reticulum (ER) and exhibit variations in the NPA motif (asparagine‐proline‐alanine) common to aquaporins (Ohta et al. 2009; Yakata et al. 2011). Super‐aquaporins are known to allow not only glycerol but also water molecules to pass through and are thought to be involved in maintaining intracellular homeostasis (Li et al. 2025). However, many of the details remain to be elucidated.

Glycerol and urea are important metabolites in the body's energy and nitrogen metabolism. Glycerol is produced by the hydrolysis of triglycerides (triacylglycerols) and serves as an energy source in the body, as well as a raw material for glucose production during gluconeogenesis. Urea is produced through the detoxification process of ammonia, which is generated during nitrogen metabolism in the body. Both glycerol and urea share common properties: they are water‐soluble, have low molecular weights, and are uncharged. This has also been confirmed in protists; experiments using [^14^C] glycerol in *Tetrahymena thermophila* have shown that glycerol is incorporated into CO_2_, the final metabolic product (Mancini et al. 2018). Furthermore, in *Tetrahymena pyriformis* , urea is produced during the early stages of the logarithmic growth phase but subsequently disappears by the time the population reaches the stationary phase; this is known to be accompanied by an increase in ammonium concentration and a change in pH (Seaman 1954).

Protists are a group of unicellular eukaryotes that inhabit diverse environments, exhibiting not only free‐living but also intestinal symbiotic and parasitic lifestyles within hosts or cells. AQPs have been reported in eight species of protists to date, and previous studies have accumulated evidence that protist AQPs, water and solute channels contribute to adaptation to changing environments. Parasitic protists that cause Chagas disease (*Trypanosoma cruzi*, Montalvetti et al. 2004; Song et al. 2014), leishmaniasis (*Leishmania* spp., Beitz 2005), malaria (*Plasmodium* spp., Hansen et al. 2002), or toxoplasmosis (*Toxoplasma gondii*, Pavlovic‐Djuranovic et al. 2003) are able to evade the immune system and establish a relatively stable environment by invading cells following infection via insect vectors (Hviid et al. 2015; Leirião et al. 2004). In human African trypanosomiasis, *T. bursei* can freely swim around in the bloodstream and obtain nutrients, mainly glucose, from the blood (Uzcategui et al. 2004; Vincent and Barrett 2015), but it is also vulnerable to attack by antibodies and immune cells (Mandal et al. 2013). In contrast, free‐living protists face harsh environments and sudden changes in osmotic conditions caused by droughts or rainfall. Examples include *Dictyostelium discoideum* (Plattner 2013; von Bülow et al. 2012, 2015), *Amoeba proteus* (Nishihara et al. 2008), and *Paramecium multimicronucleatum* (Ishida et al. 2021), likely requiring the highest levels of adaptability and responsiveness. AQP water and solute channels are localized at the cell interface with the surrounding environment and in contractile vacuoles and are thought to play a crucial role in such adaptive processes.

In a previous paper, we cloned an aquaporin‐like gene, *aqp1* from *P. multimicronucleatum*, and, by creating a fusion protein between its translated product and green fluorescent protein, demonstrated that *P. multimicronucleatum* AQP1 (PmAQP1) is localized to the contractile vacuole complex (Ishida et al. 2021). Furthermore, we showed that knockdown of AQP1 by RNAi suppresses contractile vacuole function. The predicted amino acid sequence has a six‐transmembrane structure, and its signature sequence, the NPA (Asn‐Pro‐Ala) motif, is not the typical NPA–NPA but an atypical NPA–NPG (Asn‐Pro‐Gly) sequence, and it also possesses evolutionary phylogenetic characteristics that form the same clade as super‐aquaporins (Ishida et al. 2021). These analyses suggested that this protein is involved in the water efflux mechanism mediated by contractile vacuoles; however, the specific substances that this protein allows to pass through remain unknown. The aim of this study is to express PmAQP1 in *Xenopus* oocytes, identify substances that permeate PmAQP1 using a swelling assay, demonstrate the multifunctionality of this atypical NPA–NPG channel, and investigate a new role for contractile vacuoles in free‐living protozoa—specifically, not only as organs for osmoregulation but also as pathways for the removal of metabolic waste.

## 1 Materials and Methods

### 1.1 Construction and Expression of *aqp1* mRNA and *aqp1‐Megfp* mRNA

The nucleotide sequence of the *Paramecium multimicronucleatum* AQP1 gene (AB771955) was optimized based on the codon usage of *Xenopus laevis* . The optimized sequence was synthesized by a commercial gene synthesis service (Eurofins Genomics K.K., Tokyo, Japan). A DNA fragment of PmAQP1 was isolated by PCR of sequence optimized DNA using the *EcoR* I site‐containing 5′ primer 5′‐CGCGAATTCACCATGTCCCAGGAAACTAAG‐3′ and *BamH* I site‐containing 3′ primer 5′‐CGCGGATCCTCAGTCTCCTAATTTGCTTC‐3′. To construct mEGFP‐tagged PmAQP1, PmAQP1 fragments cleaved with EcoR I and BamH I were cloned into a pT7G (UKII+)‐NL‐mEGFP transcription vector (with monomeric EGFP tag at the C‐terminus) cleaved with the same restriction enzymes (Iwao et al. 2005). A construct was cut singly at the *Not* I site, located downstream of the poly (A) tail, and then transcribed in vitro by using the MEGA Script T7 Kit (Invitrogen) with anti‐reverse cap analog (NEB).

The expression of *P. multimicronucleatum aqp1* mRNA introduced into *Xenopus* oocytes was monitored by measuring the change in fluorescence intensity caused by the expression of a PmAQP1‐mEGFP fusion protein. For fluorescence intensity analysis, fluorescence images of *Xenopus* oocytes expressing the PmAQP1‐mEGFP construct were acquired using standard fluorescence microscopy settings for mEGFP, and the resulting images were analyzed using ImageJ (https://imagej.nih.gov/ij/). Color images were converted to monochrome images (256‐level grayscale), and the number of pixels for each grayscale level was obtained from each image. Since the volume of oocytes varies between individuals, a unit area was defined within each oocyte, and the number of pixels for each grayscale level within that unit area was calculated. The average of the values from 10 individuals was calculated to determine the rate of change in brightness. This fluorescence intensity analysis revealed that the expression of the introduced mRNA reached a plateau within 48 h (Data not shown).

### 1.2 Oocyte Swelling Assay

The swelling assay using *Xenopus* oocytes was performed according to the method of Nishihara et al. (2008). Oocytes were isolated from the ovarian lobes of anesthetized adult female *Xenopus laevis* and placed in modified Barth's solution MBS (MBS: 88 mM NaCl, 1 mM KCl, 2.4 mM NaHCO_3_, 0.33 mM Ca (NO_3_)_2_·4H_2_O, 0.41 mM CaCl_2_·4H_2_O, 0.82 mM MgSO_4_·7H_2_O, 10 mM Hepes‐NaOH (pH 7.6), 10 μg/mL sodium penicillin, and 10 μg/mL streptomycin sulfate, 200 mOsm) as described by Katsuhara et al. (2002). The lobes were ruptured and treated with 1 mg/mL collagenase (034–22,363, FUJIFILM Wako Pure Chem. Co., Osaka, Japan) in Ca‐free MBS for 2 h. The isolated oocytes were washed several times and incubated in MBS for 1 day (20°C) before microinjection. 9.2 nL PmAQP1‐mEGFP mRNA (1 𝜇g/𝜇L) or deionized distilled water (DDW) was microinjected into oocyte in MBS with 4% Ficoll. 2 days after microinjection, the water permeability of oocytes was measured according to the method of Preston et al. (1992). Oocytes were photographed at regular intervals (0, 2.5, 5, 7.5, 10 min) using a digital camera (EX‐F1, Casio, Tokyo, Japan) attached to a microscope (S8APO, Leica Microsystems, Tokyo, Japan), and the changes in their major and minor axes were measured and analyzed using ImageJ. Osmotic water permeability (*P*_*f*_) was calculated from osmotic swelling data using the following formula: *P*_*f*_ = [*V*_*0*_ **×** *d*(*V/V*_*0*_)/*dt*]/[*S* **×** *V*_*w*_ **×** (osm_in_—osm_out_)], where initial oocyte volume (*V*_*0*_), relative volume (*V/V*_*0*_), surface area of oocyte (*S*), and the molecular volume of water (*V*_*w*_ = 18 cm^**3**^/mol). Oocytes were transferred from control MBS of 200 mOsm (osm_in_) to 3‐fold diluted MBS of 70 mOsm (osm_out_). For the calculation of permeability of the solutes *P*_*gly*_ and *P*_*urea*_, the molecular volume of glycerol (*V*_*gly*_ = 73 cm^**3**^/mol) and urea (*V*_*urea*_ = 45 cm^**3**^/mol) were used, respectively. For a direct comparison of water and solute permeability, the initial swelling rates (*d (V/V*_*0*_ *)/dt, /s*) were used. All data are shown with their SEM. Student's *t*‐test was used to evaluate significance.

In PmAQP1 inhibition assay, tetraethylammonium (TEA, Fujifilm Wako Pure Chemical Industries Ltd., Osaka) and phloretin (PHL, Tokyo Chemical Industry Co. Ltd., Tokyo) were used as alternatives to mercury chloride, taking into consideration toxicity and environmental impact. TEA has been reported to inhibit AQP1, AQP2, and AQP4 in the AQP expression system of *Xenopus* oocytes, and PHL is known as an aquaglyceroporin inhibitor and has been reported to inhibit AQP3 and AQP9 in the AQP expression system of *Xenopus* oocytes (reviewed by Abir‐Awan et al. 2019). All other chemicals were obtained from Sigma Aldrich Japan (Tokyo, Japan).

## 2 Results

### 2.1 Expression of PmAQP1 in *Xenopus* Oocyte

**FIGURE 1:**
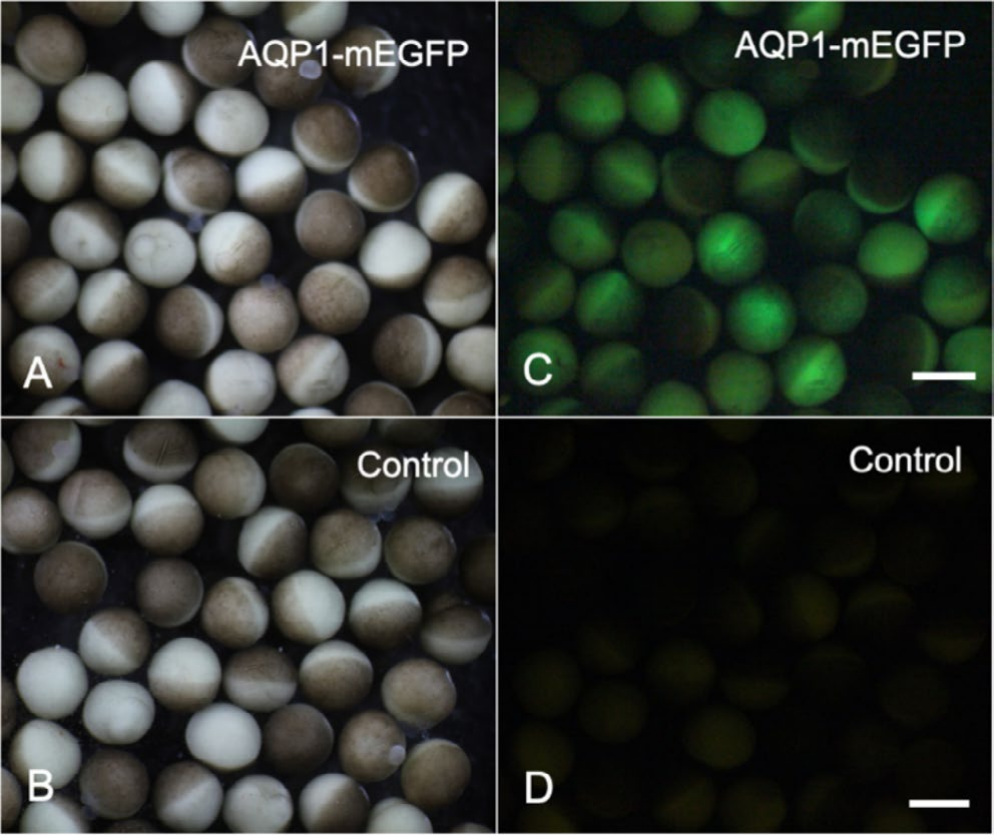
Bright‐field and fluorescence microscopy images of *Xenopus* oocytes injected with mRNA of AQP1‐mEGFP fusion protein of *Paramecium multimicronucleatum* or DDW. A and C show bright‐field images of cells injected with mRNA encoding an AQP1‐mEGFP fusion protein and their fluorescence microscopy images, respectively. B and D show bright‐field and fluorescence microscopy images, respectively, of cells injected with DDW as a control. These images were taken at 42 h after injection. Scale bar, 1.0 mm.

To investigate the expression level of PmAQP1 mRNA introduced into *Xenopus* oocytes, mRNA of a PmAQP1‐mEGFP fusion construct was synthesized, and its expression level was confirmed by monitoring changes in the fluorescence intensity emitted from mEGFP (Figure 1, A and C). For control, cells injected with DDW instead of mRNA were used (Figure 1, B and D). Figure 1 shows bright‐field images (A, B) and the fluorescence images (C, D) of *Xenopus* oocytes 42 h after injection. As indicated by the mEGFP fluorescence in Figure 1C, the introduced mRNA was translated at sufficient levels in almost all cells. Based on the average of fluorescence intensity measurements from 10 cells, the expression level of PmAQP1‐mEGFP, indicated by mEGFP fluorescence, showed sufficient fluorescence intensity as early as 18 h after post‐transfection and reached 90.8% of the observed fluorescence intensity at 42 h.

### 2.2 Functional Expression of PmAQP1 in *Xenopus* Oocytes and the Effect of Inhibitors

**FIGURE 2:**
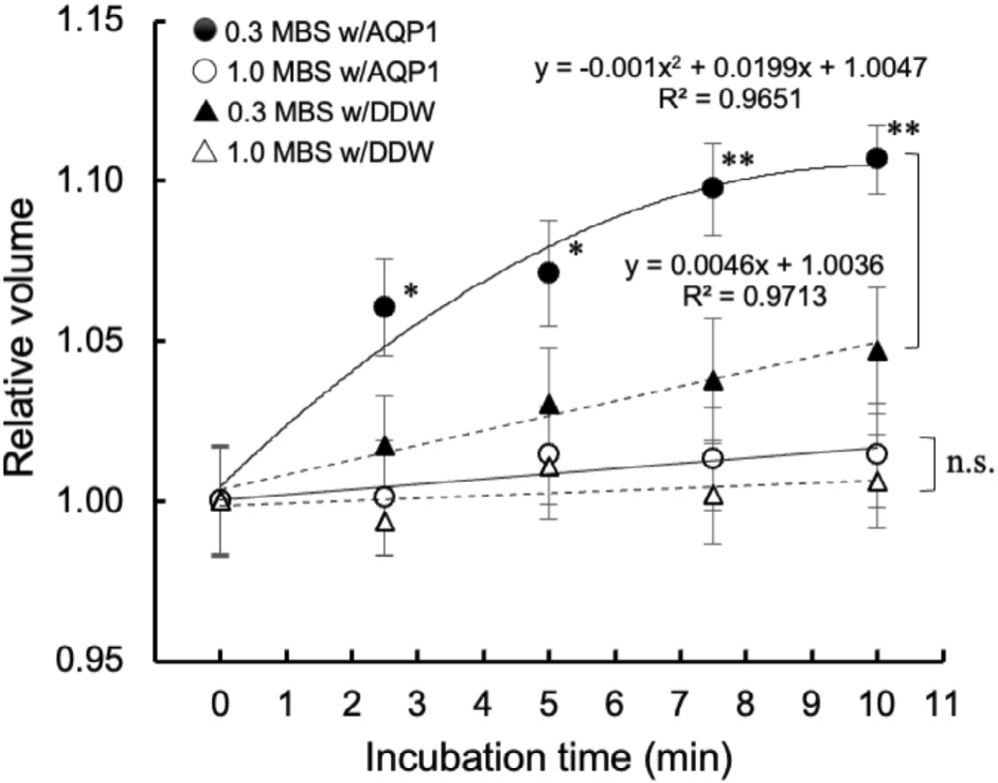
Relative changes in cell volume of *Xenopus* oocytes expressing AQP1 of *Paramecium multimicronucleatum* in hypotonic or isotonic solutions. Oocytes were injected with mRNA encoding AQP1 (open and closed circles) or DDW (open and closed triangles) as the control. Control and AQP1 expressing oocytes were transferred into a hypotonic solution (closed circles and closed triangles) or isotonic solution (open circles and open triangles) at 0 min, and monitored every 2.5 min up to 10 min. The hypotonic solution was modified Barth's solution (1.0 MBS), diluted 3‐fold with DDW (0.3 MBS). The solid line represents the quadratic polynomial approximation curve for cells injected with AQP1 in hypotonic solution, while the dashed line represents the linear approximation curve for cells injected with DDW in hypotonic solution. Equation and correlation coefficient (R^2^) of regression lines are shown immediately above the lines. Plots and error bars of the relative volume show the means and standard error of the mean (SEM) from 20 oocytes. The student's *t*‐test was performed between the closed circles and closed triangles at each time point. **, significance at *p* < 0.01; *, significance at 0.01 < *p* < 0.05; AQP, aquaporin.

Functional measurements were made in *Xenopus* oocytes after microinjection of mRNA or DDW and 48 h incubation in MBS at 20°C. Figure 2 shows the relative changes in cell volume of *Xenopus* oocytes expressing PmAQP1 in hypotonic or isotonic solution. Oocytes in isotonic solution, whether expressing PmAQP1 (Figure 2, open circle) or control cells (Figure 2, open triangle), were observed to swell only slightly during the observation period, but this was not statistically significant. After hypotonic treatment from 200 mOsm to 70 mOsm, control *Xenopus* oocytes (DDW‐injected, closed triangles in Figure 2) swelled very slowly and linearly and did not rupture within the observation period (10 min). Oocytes expressing PmAQP1 (closed circles in Figure 2) swelled relatively rapidly, with approximately 15% of the measured cells rupturing within 7.5 min and exhibiting statistically significantly higher osmotic water permeability (more than 4 times) than control oocytes (Figure 2 and Table 1). The mean and SEM for the osmotic water permeability (*P*_*f*_) of PmAQP1, calculated from measurements of 20 *Xenopus* oocytes, were 52.1 ± 9.4 (×10^−4^)cm/s (Table 1). The increased water permeability was completely inhibited by 0.1 mM tetraethylammonium (TEA), known as an orthodox aquaporin inhibitor, and 0.5 mM phloretin (PHL), an aquaglyceroporin inhibitor, reducing it to levels equivalent to or lower than that of control oocytes.

**TABLE 1:** Effects of inhibitors on osmotic water permeability of AQP1 of *Paramecium multimicronucleatum* expressed in *Xenopus* oocytes.

	External Soln.	*P*_*f*_(×10^−4^cm/s)
AQP1	0.3 MBS	52.1 ± 9.4*
	0.3 MBS—0.5 mM PHL	6.6 ± 5.4 n.s.
	0.3 MBS—0.1 mM TEA	12.5 ± 5.2 n.s.
DDW	0.3 MBS	12.3 ± 6.3

*Note:* Oocytes were injected with mRNA encoding AQP1 of *Paramecium multimicronucleatum* or DDW as a control. Osmotic water permeability (*P*_*f*_) was measured by transferring oocytes from isotonic modified Barth‘s solution (MBS) to hypotonic MBS, diluted 3‐fold with DDW (0.3 MBS) and relative volumes were obtained from measurements taken at 0 min and 2.5 min. Formulas for calculating *P*_*f*_ was given in Materials and Methods. Tetraethylammonium (TEA) or phloretin (PHL) was added to the external solution as an aquaporin inhibitor at a concentration of 0.1 mM or 0.5 mM, respectively. Bars and error bars of the relative volume show the means and standard error of the mean (SEM) from 20 oocytes.

Abbreviation: n.s., no significance.

* Significance at 0.01 < *p* < 0.05 (Student's *t‐*test).

### 2.3 Permeability for Solutes of PmAQP1

**FIGURE 3:**
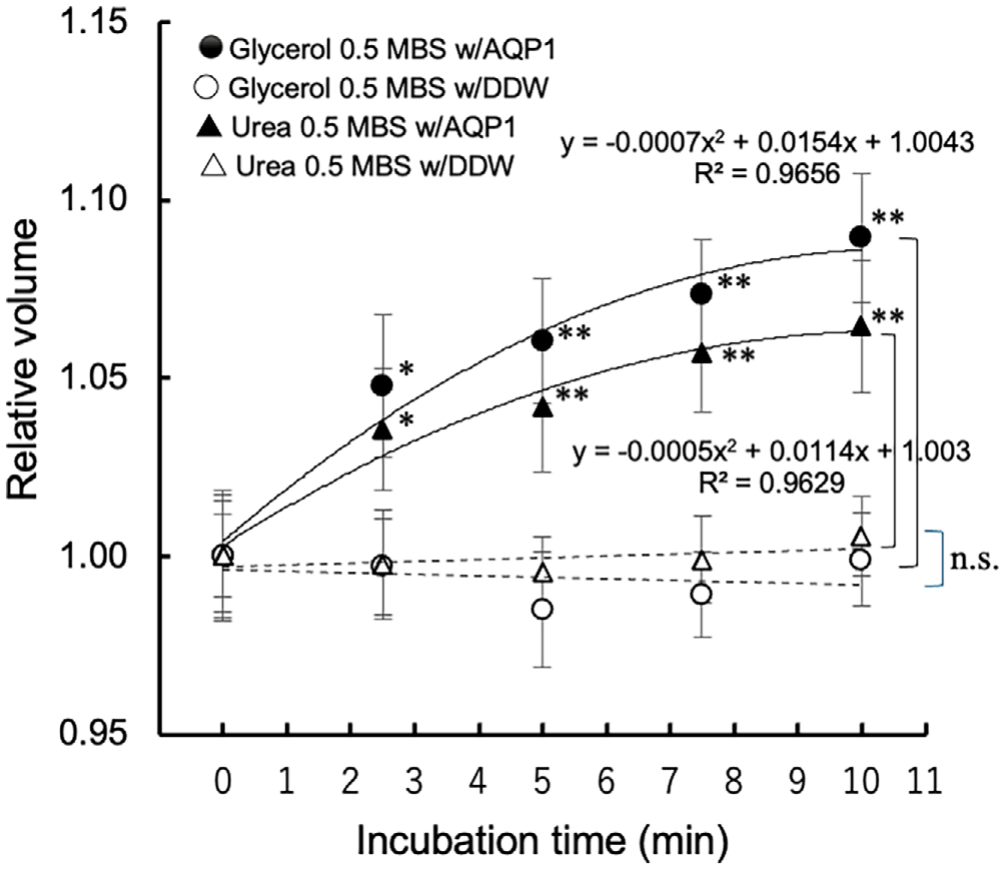
Relative changes in cell volume of *Xenopus* oocytes expressing AQP1 of *Paramecium multimicronucleatum* in isotonic solution containing glycerol or urea. Oocytes were injected with mRNA encoding AQP1 (closed circles and triangles) or DDW (open circles and open triangles) as the control. Control and AQP1 expressing oocytes were transferred into an isotonic solution containing 100 mM glycerol at 0 min, and monitored every 2.5 min up to 10 min. The isotonic solutions were prepared by adding glycerol (circles) or urea (triangles) to modified Barth's solution (MBS, 200 mOsm) diluted two‐fold with distilled water (0.5 MBS). The solid line represents the quadratic polynomial approximation curve for cells injected with AQP1. Equation and correlation coefficient (R^2^) of regression line are shown next to the line. Plots and error bars of the relative volume show the means and standard error of the mean (SEM) from 20 oocytes. The student's *t*‐test was performed between the closed circles and open circles at each time point. **, significance at *p* < 0.01; AQP, aquaporin.

The glycerol permeability of PmAQP1 expressed in *Xenopus* oocytes was analyzed using an isotonic swelling assay with a 100 mM inward glycerol concentration gradient (Figure 3, closed circle), and similarly, urea permeability was analyzed using an isotonic swelling assay with a 100 mM inward urea concentration gradient (Figure 3, closed triangle). As the control experiment, oocytes injected with DDW instead of mRNA were used in the isotonic swelling assay with a 100 mM inward glycerol gradient (Figure 3, open circle) and the isotonic swelling assay with a 100 mM inward urea gradient (Figure 3, open triangle). Oocytes expressing PmAQP1 swelled statistically significantly in an isotonic solution containing 100 mM glycerol or an isotonic solution containing 100 mM urea. From data obtained from 20 *Xenopus* oocytes during the first 2.5 min of this isotonic swelling experiment, the osmotic solute permeability to glycerol or urea was calculated. The mean and SEM values of the osmotic solute permeability of PmAQP1 to glycerol and urea were 7.0 ± 1.3 (×10^−4^)cm/s and 9.3 ± 2.7 (×10^−4^)cm/s, respectively (Table 2).

**TABLE 2:** Glycerol or urea permeability of AQP1 of *Paramecium multimicronucleatum* expressed in *Xenopus* oocytes.

	*P*_*gly*_ (×10^−4^cm/s)	*P*_*urea*_ (×10^−4^cm/s)
AQP1	7.0 ± 1.3*	9.3 ± 2.7*
DDW	0.6 ± 2.8	0.6 ± 2.4

*Note:* Oocytes were injected with mRNA encoding AQP1 of *Paramecium multimicronucleatum* or DDW as a control. Permeability to glycerol (*P*_*gly*_) and urea (*P*_*urea*_) was measured in isotonic conditions by adding 100 mOsm glycerol or 100 mOsm urea to a 2‐fold dilution modified Barth's solution with DDW and relative volumes were obtained from measurements taken at 0 min and 2.5 min. Formulas for calculating *P*_*gly*_ or *P*_*urea*_ is described in Materials and Methods. Bars and error bars of the relative volume show the means and standard error of the mean (SEM) from 20 oocytes.

* Significance at 0.01 < *p* < 0.05 (Student's *t‐*test).

**FIGURE 4:**
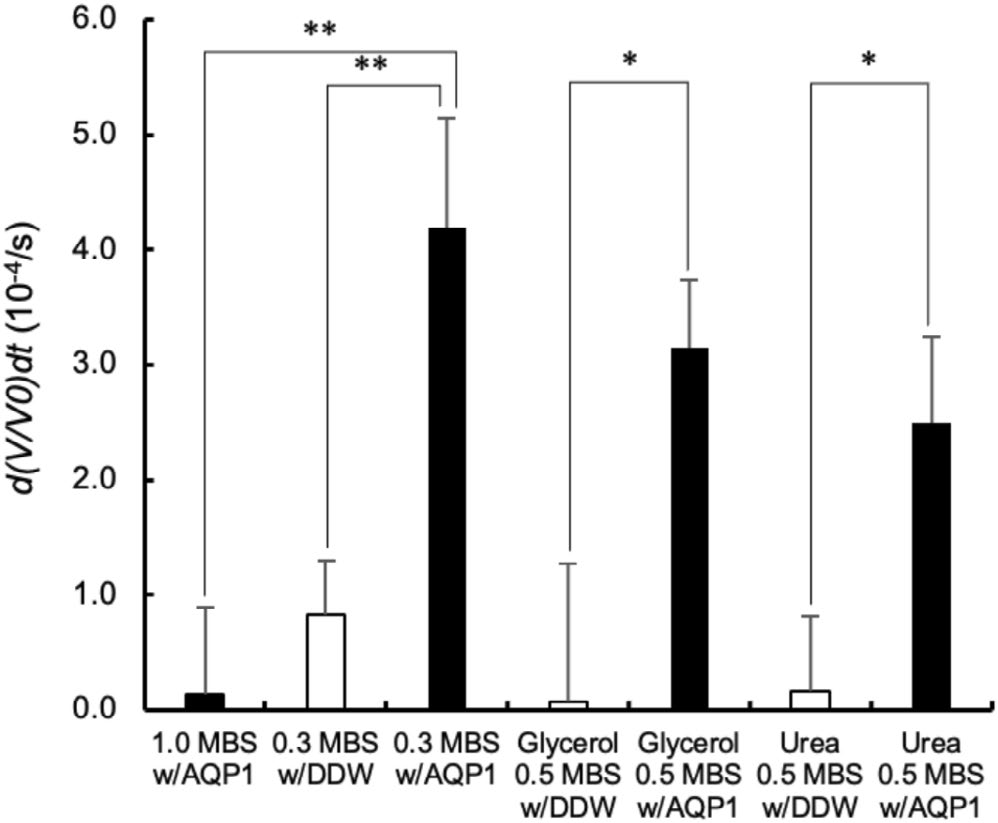
Swelling rates of *Xenopus* oocytes expressing or not expressing AQP1 derived from *Paramecium multimicronucleatum*. Oocytes were injected with mRNA encoding AQP1 (solid bars) or DDW (open bars) as the control. Control and AQP1 expressing oocytes were transferred into a hypotonic solution (0.3 MBS), isotonic solution (1.0 MBS), or isotonic solutions (0.5 MBS) containing 100 mM glycerol or 100 mM urea. Relative volume changes were calculated from measurements taken at 0 min and 2.5 min. Bars and error bars of the relative volume show the means and standard error of the mean (SEM) from 20 oocytes. The student's *t*‐test was performed between the bars connected by solid lines. **, significance at *p* < 0.01; *, significance at 0.01 < *p* < 0.05; ns, not significant; AQP, aquaporin.

To compare the permeability of water and solutes in *Xenopus* oocytes expressing PmAQP1, we used initial swelling rate (*d (V/V*_*0*_ *)/dt*) from the start of osmotic treatment up to 2.5 min, and the results were shown in Figure 4. PmAQP1 exhibited permeability to glycerol and urea, following that of water, and its initial swelling rates showed significant differences compared to the control in all cases.

## 3 Discussion

### 3.1 Expression of PmAQP1 in *Xenopus* Oocytes

As shown in Figure 1, PmAQP1 mRNA introduced into *Xenopus* oocytes showed sufficient fluorescence intensity 2 days after intracellular injection (Figure 1). In the hypertonic swelling assay used to examine water permeability (Figure 2), changes in the rate of cell swelling corresponding to changes in expression levels over time were recorded (data not shown), and in the isotonic swelling assay used to examine the permeability of glycerol and urea (Figure 3). Cell swelling was confirmed in both cases. In addition, experiments using inhibitors (Table 1) also showed that the introduced PmAQP1 was inhibited. These results suggest that the PmAQP1 introduced into the cells was indeed expressed and expressed on the cell membrane of *Xenopus* oocytes.

### 3.2 Water Permeability of PmAQP1

In hypotonic swelling experiments, *Xenopus* oocytes expressing PmAQP1 showed a statistically significant swelling rate of more than four times compared to control oocytes injected with DDW. The *P*_*f*_ calculated from measurements of 20 oocytes is approximately 50 (×10^−4^)cm/s. This permeability rate is close to the value reported by Sugino et al. (2005) for contractile vacuole membranes isolated under a microscope from *Paramecium multimicronucleatum*, close to the value reported by Nishihara et al. (2008) for the water permeability of ApAQP distributed in the contractile vacuole of *Amoeba proteus* , and almost identical to the value reported by von Bülow et al. (2012) for the water permeability rate of AqpB mutants (Δ208–219) distributed in the contractile vacuole of *Dictyosterium discoideum*, suggesting that it is a relatively slow water channel. Table 3 shows the AQP observed in protists whose water or solute osmosis rates were measured and reported in the literature. Among the AQPs of the seven protozoan species shown in Table 3. Those distributed in contractile vacuoles or vacuolar structures associated with contractile vacuoles tend to have relatively slow water permeation rates, whereas those distributed on the cell membranes or flagellar membranes of parasitic protozoa appear to have very fast rates.

**TABLE 3:** Water and solute permeability of AQPs in protists.

	AQPs	Localization	*P*_*f*_ (×10^−4^cm/s)	*P*_*gly*_ (×10^−4^cm/s)	*P*_*urea*_ (×10^−4^cm/s)	References
*Amoeba proteus*	ApAQP	contractile vacuole	65 ± 17	n.d.	n.d.	Nishihara et al. (2008); von Bülow and Beitz (2015)
*Toxoplasma gondi*	TgAQP	contractile vacuole	37 ± 2.0	0.36 ± 0.08	0.33 ± 0.10	Pavlovic‐Djuranovic et al. (2003); Beitz (2005); Zeuthen et al. (2006); von Bülow and Beitz (2015)
*Trypanosoma cruzi*	TcAQP	contractile vacuole	32 ± 9	2.3 ± 0.01	n.d.	Montalvetti et al. (2004); Beitz (2005); von Bülow and Beitz (2015)
*Dictyostelium discoideum*	AqpB, Δ208–219	plasma membrane, vacuolar structures	51	Non‐pass	Non‐pass	von Bülow et al. (2012); von Bülow and Beitz (2015)
*Plasmodium falciparum*	PfAQP	plasma membrane	276	14 ± 0.1	4.0 ± 0.1	Hansen et al. (2002); Beitz et al. (2004); Zeuthen et al. (2006); von Bülow and Beitz (2015)
*Leishmania major*	LmAQP1	flagellm	180	0.8	pass	Figarella et al. (2007)
*Trypanosoma brucei*	TbAQP	flagellar membrane	150–180	pass	pass	Uzcategui et al. (2004); von Bülow and Beitz (2015)

Abbreviations: n.d, not determined; *P*_*f*_, water permeability; *P*_*gly*_, glycerol permeability;*P*_*urea*_, urea permeability.

The parasitic protists shown in Table 3 (*Toxoplasma gondii, Trypanosoma cruzi, Plasmodium malariae, Leshmania major, Trypanosoma bursei*) inhabit relatively stable environments such as insect vectors, host fluids, or cytoplasm. On the other hand, free‐living protists such as *Amoeba proteus* and *D. discoideum* encounter significant changes in osmotic conditions due to rainfall, drought, etc. Interestingly, AQPs with very fast water permeability observed in parasitic protists have not been reported to be distributed in contractile vacuoles. On the other hand, those reported to be distributed in contractile vacuoles have relatively slow water permeability (Table 3, von Bülow and Beitz 2015).

If we assume that free‐living protists express highly permeable AQP on their cell membranes, then changes in external osmotic pressure would immediately affect the cell body via AQP. In such extremely hypotonic environments, it would be necessary to express highly permeable AQP in the contractile vacuole to rapidly remove water from the cell body to avoid cell rupture. However, there are no reports of such highly permeable AQP being distributed in the contractile vacuole (Table 3, von Bülow and Beitz 2015). On the other hand, while there are reports that slow‐permeable AQP is distributed in the contractile vacuole, there are no reports of it being distributed in the cell membrane except for *D. discoideum* (Table 3, von Bülow and Beitz 2015). Wild‐type AqpB of *D. discoideum*, which is also distributed in the cell membrane, is impermeable to water, but the mutant Δ208–219, which has a cleaved intracellular loop, has been reported to be permeable to water, suggesting that it may be regulated via the intracellular loop (von Bülow et al. 2012).

Based on this, we speculate that organisms living in extremely harsh environments may not express AQPs on their cell membranes, or if they do, the AQPs may be in an inactive state. According to Stock et al. (2001). The water permeability of the cell membranes of the free‐living protists *P. multimicronucleatum* and *A. proteus* is reported to be close to that of the oocytes of the *Xenopus* oocytes. Similarly, in the green alga *Chlamydomonas reinhardtii* , the water permeability of the contractile vacuole is reported to be 42–101 (×10^−4^)m/s, even though the water permeability of the cell membrane is at a level where aquaporins are absent (Komsic‐Buchmann et al. 2014). However, to date, only eight species of protists, including *Paramecium*, have been confirmed to possess AQP, so further evidence is needed to advance this argument.

### 3.3 Solute Permeability of PmAQP1

In the isotonic swelling assay of *Xenopus* oocytes, the extracellular solution was isotonic, yet contained 100 mM glycerol or 100 mM urea, and was designed to create an osmotic gradient of 100 mM solute towards the inside of the cell. Under these conditions, cell swelling begins when glycerol or urea flows into the oocyte due to the concentration gradient between the inside and outside of the cell. The influx of glycerol or urea into the cell increases the osmotic pressure of the cytoplasm, leading to a secondary influx of water. Therefore, in the case of swelling caused by the influx of glycerol or urea, the water permeability of PmAQP1 acts as the limiting factor. In fact, the rate of swelling caused by these solutes never exceeded the rate of swelling resulting from the water permeability of PmAQP1 (Figure 4).

### 3.4 Limitation of the Study

This study has two main limitations that should be acknowledged. First, we did not morphologically confirm the localization of PmAQP1 in the plasma membrane of *Xenopus* oocyte using tomographic analysis such as confocal laser microscopy. However, the significant volume changes observed in both hypotonic and isotonic swelling assays, combined with the complete inhibition of water permeability by specific inhibitors (TEA or PHL), provide strong functional evidence that PmAQP1 is properly integrated and functioning at the cell surface. Secondly, it was not possible to accurately determine the true maximum permeability rates of glycerol and urea. Because the water permeability of PmAQP1 is relatively slow, water influx becomes the rate‐limiting step during solute‐induced swelling. As demonstrated in Hansen et al. (2002), current experiments also conclude that it is impossible to measure the true permeation rate of these solutes via PmAQP1 unless a channel that permeates water faster than PmAQP1 and does not allow glycerol or urea to pass through (e.g., RatAQP1 or HsAQP1) is co‐expressed. Therefore, the solute permeation rates reported here are likely underestimated. Despite these limitations, our current data unequivocally demonstrate that PmAQP1 possesses the ability to permeate water, glycerol, and urea.

### 3.5 Physiological Significance of PmAQP1 Multifunctionality

As Patterson (1980) points out in his extensive review, “Griffith (1888) presented evidence that nitrogen metabolic waste products are excreted from protozoa via the contractile vacuole, but Weatherby (1941) and Kitching (1967) both found the evidence supporting this function unconvincing,” highlighting the long‐standing debate over whether nitrogen metabolic products are released from the contractile vacuole. In this regard, Heuser et al. (1993) proposed the hypothesis that metabolites such as ammonia and bicarbonate are osmoregulatory substances that can be continuously removed as osmotic substances. Furthermore, Rohloff and Docampo (2008), based on experimental data from the parasite *T. curuzi*, suggested that contractile vacuoles may also have the function of releasing metabolites in addition to osmoregulation. However, to date, there is no direct evidence that these end‐products of metabolism are contained within contractile vacuoles.

Addressing this long‐standing debate, our current research reveals a noteworthy discovery: PmAQP1, localized in the contractile vacuole of the free‐living protozoan *Paramecium*, transports urea. Glycerol and urea have also been reported to be utilized in energy and nitrogen metabolism in protists (Mancini et al. 2018; Seaman 1954). Regarding urea, since it acts as a protein denaturant and is harmful at high concentrations, it is highly likely to be excreted into the extracellular space via the contractile vacuole (von Bülow and Beitz 2015). As summarized in Table 3. There have been no previous reports of AQPs in free‐living protozoa that transport urea, making this discovery an important functional link.

PmAQP1 also facilitates the passage of glycerol, but its physiological role remains unclear. In yeasts (Hohmann 2002) and *Caenorhabditis elegans* (Lamitina et al. 2004). It is known that glycerol biosynthesis is induced as a physiological response to hyperosmolarity treatment. This glycerol then functions as an intracellular osmolyte, increasing intracellular osmotic pressure and allowing the cells to adapt to a hyperosmolarity state. However, there have been no previous reports on the increase in intracellular glycerol concentration in response to hyperosmolarity treatment in *Paramecium*, and therefore it is not possible to discuss whether glycerol functions as an osmolyte in adaptation to hyperosmolarity solutions.

While further In Vivo studies are required to fully elucidate the physiological dynamics of these solutes in *Paramecium*, our current findings provide the first functional evidence regarding the multifunctionality of this atypical NPA‐NPG channel. Ultimately, this study proposes a new hypothesis: the contractile vacuole of *Paramecium* functions not only as a water‐extraction pump for osmotic regulation but also as a highly sophisticated mechanism for removing metabolic waste products.

## Funding

Japan Society for the Promotion of Science (16H02896, 21A402, 26K09269, JP22H05698, JP23K18485, JP24H01497, JP25H01156).

## Data Availability

The data that support the findings of this study are available on request from the corresponding author. The data are not publicly available due to privacy or ethical restrictions.
